# Kitesurfing and snowkiting injuries in Norway: a retrospective study

**DOI:** 10.1186/s13102-024-00812-w

**Published:** 2024-01-22

**Authors:** Venke Torland, Øyvind Thomassen, Øyvind Østerås

**Affiliations:** 1 Dept of Anaesthesia and intensive Care, Stavanger University Hospital, 4068 Stavanger, PO Box 8100, Norway; 2 Department of Anaesthesia and Intensive Care, Haukeland University Hospital, 5021 Bergen, PO Box 1400, Norway; 3 Mountain Medicine Research Group, University of Bergen, 5020 Bergen, PO Box 7804, Norway; 4 Norwegian Air Ambulance Foundation, PO Box 414 Sentrum, 0184 Oslo, Norway

**Keywords:** Kite, Kitesurfing, Snowkiting, Injury severity, Athletic injuries, Norway

## Abstract

**Background:**

Kiteboarding (kitesurfing on water and snowkiting) is a fairly new sport and is defined as a high-risk sport. The injury rate has been reported to be between 6 and 9 per 1000 h. The aim of the study was to identify and describe kiteboarding-related injuries in Norway over a five-year period.

**Methods:**

We used “snowball sampling” to identify kiteboarding accidents in a retrospective study. In addition, we conducted structural searches in the National Air Ambulance Service and Search and Rescue Helicopter patient record databases. All included informants were interviewed. Descriptive methods were used to characterise the sample.

**Results:**

Twenty-nine kiteboarders were included, with a total of 33 injuries. One half of the injuries to head, face and neck were cerebral concussions (*n* = 12). The most common type of injury was bone fractures (*n* = 28), followed by soft tissue injuries (*n* = 24). Most injuries were of moderate severity (51%) and falling from less than 5 m was the most common mechanism of injury. Operator error and lack of experience were the most frequently reported causes of accidents (82%).

**Conclusions:**

Serious injuries occured during kiteboarding. The majority of kiteboarders reported operator error or lack of experience as the cause of their accident. Prior to kiteboarding, a course highlighting the importance in using helmet for snowkiting and both helmet and life vest in kitesurfing, should be mandatory.

**Supplementary Information:**

The online version contains supplementary material available at 10.1186/s13102-024-00812-w.

## Background

**Fig. 1:**
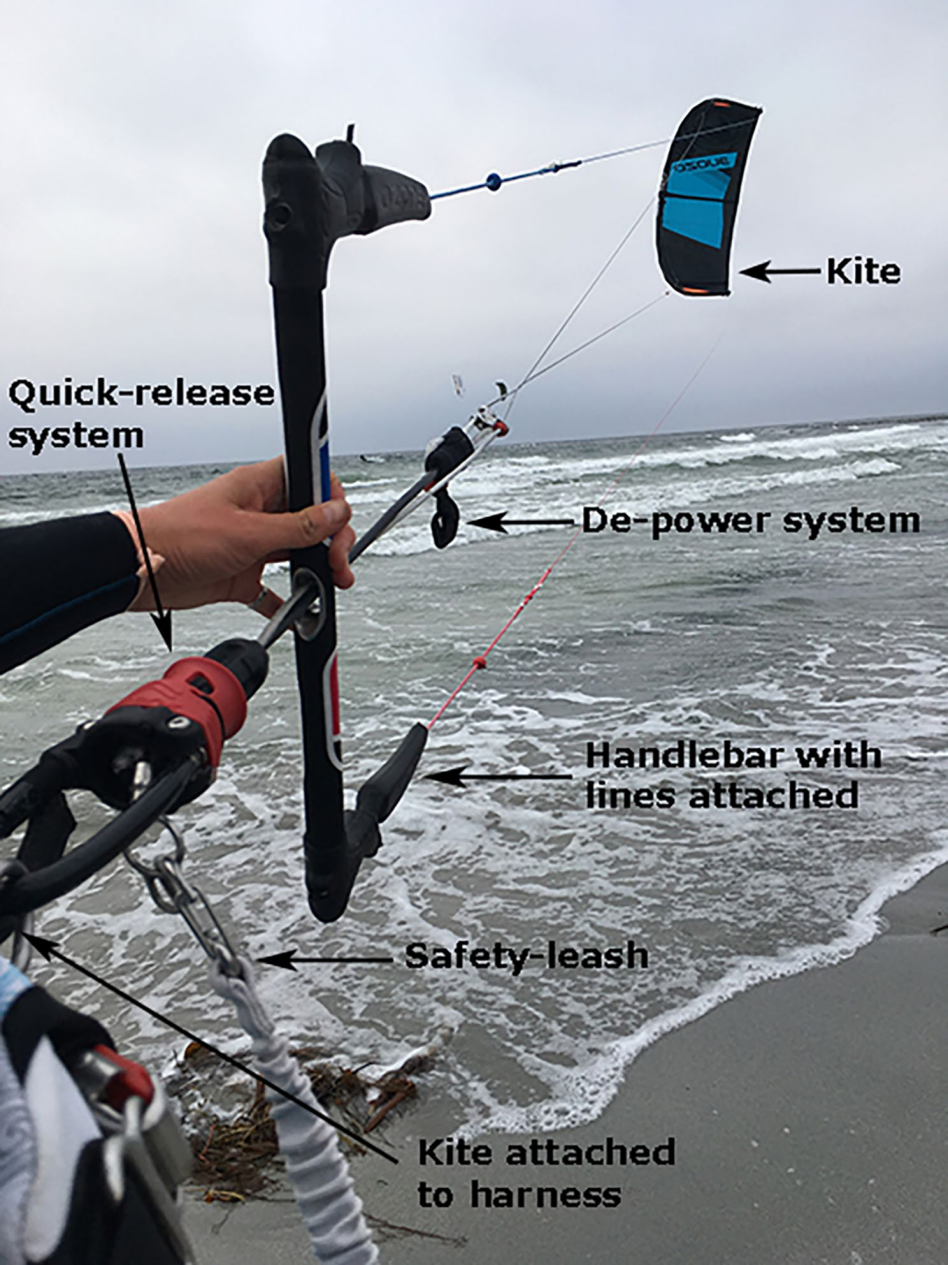
Picture of a kite illustrating the depower and quick-release systems

Kiteboarding (referred to as kitesurfing on water and snowkiting on snow) is a fairly new sport. In kiteboarding, a kite is used to achieve wind power and speed on water or snow (Fig. 1). On water, a small twin-tip board, surfboard, or raceboard is used to navigate the surface. Skis or snowboards can be used on snow.

Kiteboarding can be defined as a high-risk sport [1]. Kiteboarders can achieve speeds on snow exceeding 100 km/h, and can make jumps as long as 1000 m. These are activities that present an obvious risk for injury [2]. Operator errors, unstable and shifting winds, and poor snow conditions are all recognised as common factors that contribute to an increased risk of injuries [1]. In the past decade, all new kites have been equipped with a depower system, allowing kiters to regulate the traction of the kite while kiteboarding. In addition, a quick-release system allows the kiteboarder to reduce most of the power of the kite in case of an emergency while still being attached to the kite. Today, most kiteboarders (95%) use equipment that has this quick-release system [1, 3].

Studies from Australia and New Zeeland have documented injury rates ranging from 5.9 to 7 per 1,000 h in kitesurfing [3, 4]. An Austrian study documented an injury rate of 8.4 per 1,000 h in snowkiting [5]. The most common injuries are contusions, abrasions, lacerations, and muscle strain, but multitrauma, strangulations, hypothermia, and deaths have been reported [6–9]. A growing number of articles describe kiteboarding accidents on water. However, less is known about kite accidents on snow and the severity of these accidents are still sparsely described. More knowledge on the severity of injuries, mechanism of injuries and injury patterns is necessary for both preventive measures and for optimal emergency medical treatment. The level of experience may alter the risk of being exposed to an accident. The main aim of this study was to identify and describe kiteboarding-related injuries in Norway over a five-year period. The secondary aim was to gather an overview of the causes of the kite accidents and possibly preventable measures.

## Methods

We conducted a retrospective study using a chain-referral sampling, snowball sampling, to identify kiteboarding accidents [10]. During February 2015, we invited kiteboarders to report all accidents experienced in Norway from 2010 to 2014, either involving themselves or accidents witnessed. This invitation was sent to the seven Norwegian kiteboarding clubs, the Norwegian kiteboarding association, two kiteboarding course organisers in Norway (Fluid and URGE), and, at the time, the most visited kiteboarding webpage in Norway (www.hangloose.no). The information on the webpage had 1,781 individual views. We urged the seven kite clubs to invite their 3,967 members and sent an invitation via e-mail using contestant lists from kiteboarding competitions. Posters were positioned at Haugastøl, Haukeliseter, and Finse, which are three popular snowkiting locations. In addition, 156 personal invitations were sent by mail, including a request to forward the mail to other possible informers. Additionally, all 20 air ambulance services and the six Search and Rescue Helicopter bases in Norway performed a predefined, structural search for “kite” in their medical records.

### Participants

All kiteboarders who had experienced a kite accident occurring in the five-year period, either identified by reports or through the search in the air ambulance medical journal, were contacted to obtain informed consent. The consent forms were signed and returned to the primary investigator. Participants that did not sign a declaration of consent, and accidents happening abroad, were excluded.

### Variables/data sources

All kiteboarders were interviewed by one of the authors (VT) to report the temperature, wind, light, and snow conditions at the time of the accident. We registered the participants’ gender and age and asked them to estimate their total pre-accident exposure time, which was defined as the total number of kiteboarding hours before the accident. The mechanism of injury, anatomic region of injury, and type of injury were also registered. When available, data were cross-checked with medical records from the air ambulance services. We also interviewed the participants about the location of the accident, safety equipment used, and whether the kiter had participated in a certified kiteboarding course. The severity of the injury was registered using NACA score [see Additional file 1] and Injury Severity Score (ISS) [11, 12]. In the accidents involving the Air Ambulance Service, the registered NACA score from the medical record was used. Otherwise, injuries were retrospectively scored independently by two authors (ØT, ØØ) based on available information. Any discrepancies in retrospective NACA scoring were discussed until an agreement was reached. Abbreviated Injury Scale (AIS) and ISS were retrospectively scored (according to Abbreviated Injury Scale [AIS 2005, updated in 2008] by an authorised AIS scorer [ØØ]) by assessing the patients’ hospital records or, if not available, by the kiteboarder’s description of the injury [13]. AIS was reported for each individual injury in all accidents and used to calculate the ISS for each injury.

### Statistical methods

We used descriptive methods to characterise the sample. Normally distributed data are presented as the mean and standard deviation (SD); otherwise, medians and ranges are presented. Data were analysed with IBM SPSS Statistics for Windows, Version 23 (IBM Corp., Armonk, NY, USA). The relationship between the severity of the injuries and the kiteboarders’ experience level was analysed by Fischer’s exact test.

## Results

**Fig. 2:**
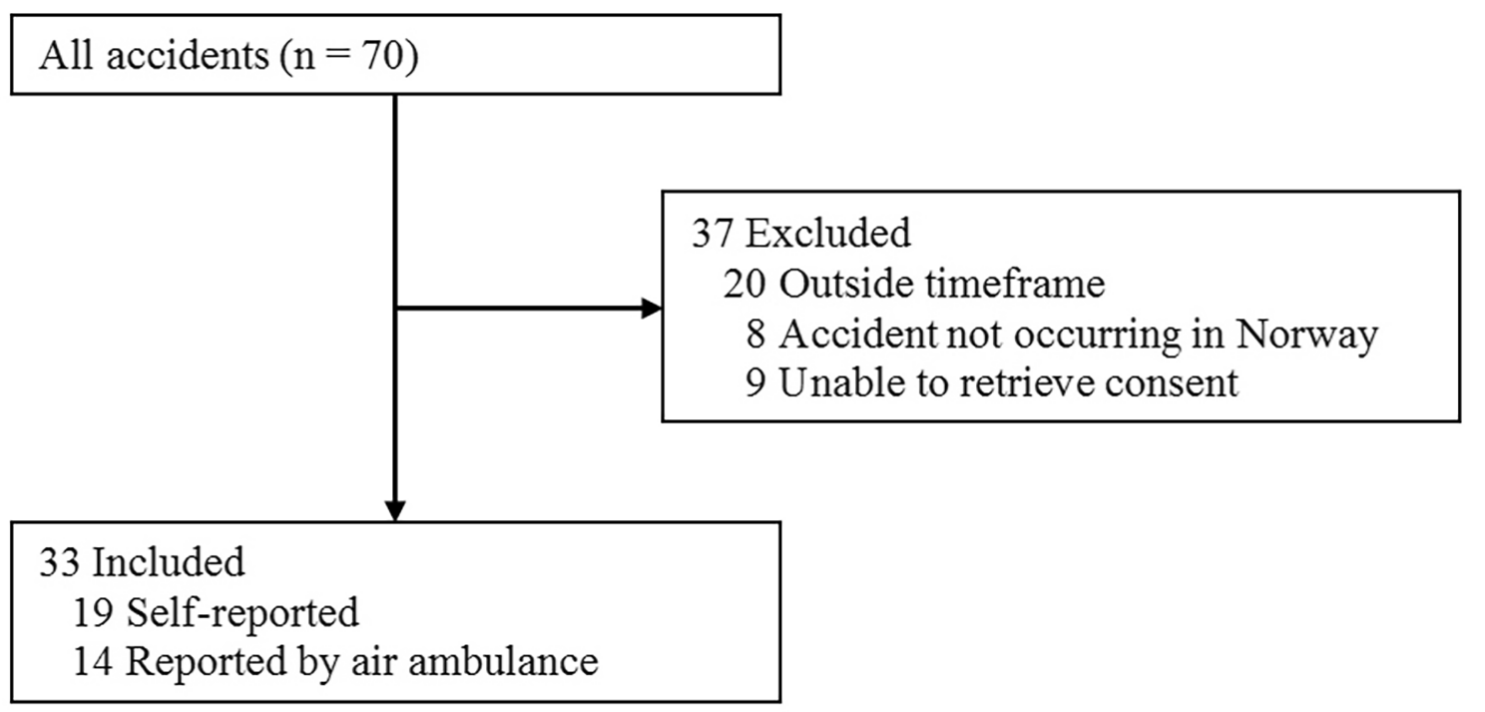
Flow chart showing the total number of accidents identified and excluded and included accidents

The median age of the participants was 33 years (range, 17–62), and 26 (90%) were males. Two of the responders came from outside Norway. Of the identified kiteboarding accidents, 29 kiteboarders experienced 33 accidents (Fig. 2). Of the 33 accidents, 18 (55%) occurred during snowkiting, and 15 occurred on water.

In the accidents during snowkiting, skis were used in 14 accidents, and a snowboard was used in 4 accidents. The estimated median preinjury experience in kiteboarding was 180 h (range, 0–3,000 h).

The most frequent body regions injured were the head, face, and neck, followed by the lower and upper extremities (Table 1). One half of the injuries to head, face and neck were cerebral concussions. The most common type of injury was bone fractures, followed by soft tissue injuries.

**Table 1:** Injury pattern and severity of 69 injuries in 33 accidents, based on AIS ^a^

		Bone fractures	Soft tissue	Cerebral concussion	Other injuries	SUM Body region		AIS		
								1	2	3
Head, face, and neck		5	6	12	1	**24**		7	17	
Thorax		6			3	**9**		2	1	6
Abdomen					1	**1**			1	
Back (spine)		2	1			**3**		1	1	1
Upper extremity		7	7			**14**		6	7	1
Lower extremity		8	10			**18**		8	8	2
SUM		**28**	**24**	**12**	**5**	**69**		**24**	**35**	**10**

^a^ Abbreviated Injury Scale 2005, updated in 2008 [13].

Most injuries were of moderate severity (AIS 2). In our patient population, 9 accidents had an ISS score of more than 9, the highest being 22. The ISS, injuries, and description of these accidents are presented in Table 2.

**Table 2:** Accident and injury descriptions in accidents with Injury Severity Score (ISS) > 9

Surface	ISS	Accident description	Injury
Snow	12	Lifted by kite towards a rock. Most likely hit the rock with the head. The kite got stuck in a mast and the kiteboarder was left hanging unconscious above ground.	Cerebral concussion and unconsciousness, 2 fractures in os. zygomaticus/mandibula, lacerations in the face, and scaphoid fractures.
Snow	12	Fall while jumping 10–15 m. Flat light in the landing.	Neck fracture of the humerus and cerebral concussion, and 2 fractured ribs.
Water	13	Miscalculated the wind direction when jumping 5–6 m over a pier. Landed on a jetty.	4 back fractures, hematoma compressing the spine causing a temporarily reduced sensibility, 1 tarsal ligament tear, and abdominal, gluteus, and back muscle tears.
Snow	13	Lost control during kiteboarding and got dragged into a parked motor vehicle.	Clavicular fracture, 9 costa fractures, and lung contusion.
Snow	13	Falling 5 m after wind gust on snow. Landed with spread legs.	Open book fracture in the pelvis, paralyzed leg, cerebral concussion, and hypothermia.
Snow	14	Falling 7 m vertically and 15 m horizontally after a wind gust. Landing on ice.	Fracture of all costae on the right side, compression fracture in shoulder and hips, facial laceration, and contusion in one arm.
Snow	14	Caught by whirlwind and thrown 4–5 m vertically and 150 m horizontally.	Unconscious for short time, cerebral concussion, rib fracture, pneumothorax, soft tissue injury in thigh and shoulder, and facial laceration.
Water	17	Lost control of the kite in a strong wind. The kite inverted and started looping. Crashed on rocks.	Unconscious for short time, open jaw fracture, mandibular fracture, maxillary fracture, 5 rib fractures, contusion of n. opticus, and facial lacerations.
Snow	22	Lifted 10–20 m vertically and 50 m horizontally by the kite on snow.	Femur fracture, 2 pelvic fractures, columnar compression fracture, and hematoma in lung and kidney.

The level of experience of the respondents and the severity of injuries were not related (*p* > 0.05). 52% had attended a kiteboarding course prior to the accident. A varying degree of safety equipment was used (Table 3). Quick-release system and safety leash are illustrated in Fig. 1.

**Table 3:** Safety equipment used in 33 accidents

	Total (*n* = 33)		Water (*n* = 15)		Snow (*n* = 18)	
Safety equipment	**n**	**(%)**	**n**	**(%)**	**n**	**(%)**
Quick-release system	32	(97)	15	(100)	17	(94)
Safety leash	32	(97)	15	(100)	17	(94)
Helmet	21	(64)	4	(33)	17	(94)
Spine protection	6	(18)	-	(-)	6	(33)
Life vest	4	(12)	4	(33)	-	(-)
Knee protection	1	(3)	-	(-)	1	(6)

The average estimated mean wind strength at the time of the accidents was close to 10 m/s. Operator error and lack of experience were most frequently reported as the only or one of the assumed reasons for the accident (Table 4).

**Table 4:** The reported causes of 33 accidents

Reported causes of accidents	Total (*n* = 33)		Water (*n* = 15)		Snow (*n* = 18)	
	n	(%)	n	(%)	n	(%)
Operator error /lack of experience	27	(82)	15	(100)	12	(67)
Difficult wind conditions	11	(33)	2	(13)	9	(50)
Wind strength	8	(24)	3	(20)	5	(28)
Snow conditions	5	(15)	0	(0)	5	(28)
Fatigue	3	(9)	2	(13)	1	(6)
Other kiters	1	(3)	0	(0)	1	(6)
Light conditions	1	(3)	0	(0)	1	(6)
Inability to detach from kite	1	(3)	1	(6)	0	(0)

Among the operator errors reported, ten were jump-related, nine resulted from misjudging strong winds, and two each stemmed from kitesurfing too close to shore or losing kite control. Wind gusts were reported as a contributing factor in 8 of the accidents. Problems with the kite equipment were not reported. Falls from less than 5 m were the most common mechanisms of injury (Table 5).

**Table 5:** Mechanism of injury, number injured, surface where injury occurred, and NACA and ISS scores

Mechanism	N (%)	Surface	NACA Median (range)	ISS Median (range)
Fall < 5 m	12 (36%)	5 snow 6 water 1 land ^a^	3 (1–4)	4 (1–14)
Fall > 5 m	10 (30%)	8 snow 2 water	3.5 (3–5)	12 (4–22)
Collision	8 (24%)	4 snow 4 water	3 (1–4)	4 (1–17)
Drowning ^b^	2 (6%)	2 water	2 (0–4)	0
Injured by kite equipment	1 (3%)	1 snow	2	0

^a^ On ground, during preparation for kiting ^b^ Nonfatal drowning

## Discussion

The severity of the reported kiteboarding injuries varied from minor lacerations to severe multitrauma. Injury severity is only sparsely described in other papers on kiteboarding, making comparisons difficult. The AIS scores for the injuries described here, where more than half resulted in moderate to severe injuries, demonstrates the possibility for harm for kiteboarders.

The proportion of cerebral concussions was in accordance with three previous studies on snow and water [1, 5, 14]. However, compared with these three reports, we also found a high number of fractures. Other high-risk sports reporting extremity fractures and cerebral concussion as the most common injuries are paragliding and hang-gliding [15, 16]. However, unlike paragliding and hang-gliding, where spinal injuries are also common, this study included only two spine fractures. This discrepancy might be due to the comparatively lower altitudes typically involved in kiting jumps.

The median ISS score was 12 in accidents resulting from falls from 5 m or more, indicating the most severe mechanism of injury in our data. However, one of the multitrauma cases with an ISS score of 17 was caused by a collision, and another injury had an ISS score of 14 after a fall from less than 5 m. Hence, life-threatening injuries may occur from kiteboarding accidents by different mechanisms of injury. When treating patients experiencing a kiteboarding accident, the many mechanisms of injury possible in kiteboarding accidents should be kept in mind. Severe injuries occurred both on snow and on water. ISS was scored retrospectively and does not necessarily reflect threats to life in the individual accident. Obviously, an injury occurring on water may be an immediate threat to life, especially if a kitesurfer is not wearing a life vest.

Although 94% used a helmet on snow, only 33% used a helmet on water. Nevertheless, helmet use was more frequent than an earlier study reporting only 4%, by van Bergen et al. in 2020 [14]. Only 2 of the 12 cerebral concussions in our study occurred on water, but there is a risk of head injuries from boards, other kiteboarders, rocks, obstacles, and falls from heights in kitesurfing. The use of helmets should be emphasised. We report one kitesurfer experiencing a nonfatal drowning and another rescued by the Air Ambulance while being pulled offshore by the wind. None of these kitesurfers wore a life vest, and only four out of 14 of the injured kiteboarders used a life vest. This low use of a life vest is in accordance with an article from 2005 showing that none of the 30 rescued kiteboarders in Cape Town wore a life vest [8].

We found a higher proportion of injured athletes who attended a kiteboarding course prior to the accident (52%) compared with an Austrian study (17.5%) [5]. One-third of the accidents occurred when the wind speed was more than 10 m/s. Moroder et al. found 18.2% [5]. These results demonstrate that many accidents occur during low wind speeds. Moroder et al. also proposed the onset of a sudden wind gust as a contributing cause of accidents, as reported in our study. Increased knowledge on wind patterns acquired during a kiteboarding course may help to avoid accidents resulting from wind gusts. Operator error, lack of experience, and wind conditions have been described previously as the most common reasons for kiteboarding accidents [5, 14]. In contrast, we found no difference in injury severity related to experience level. Nevertheless, in our study, four out of five participants cited operator errors or lack of experience as a contributing factor to the accident. Experience in this activity could indeed play a key role in avoiding accidents.

A recall bias may be present in our retrospective design, especially regarding data on weather conditions and the estimated preexposure time. Although the accidents happened some years ago, the data collection and interviews were performed in 2015. The injury severity was scored by one of the authors and, in some cases, based on information provided retrospectively by patients and their knowledge and understanding of their injury. This might have led to an under- or overestimation of NACA and ISS scores. The reported causes of the accidents and estimation of the wind strengths were based on the kiteboarders subjective assumptions, which can be difficult to state accurately. The snowball sampling method has limitations in identifying accidents because an unknown proportion of kiteboarders are members of a kiteboarding club, and no licence or a registered course is mandatory before performing the sport. It is likely that the method more readily identified the most severe injuries, potentially leading to the omission of some minor injuries in the reporting. This discrepancy might have distorted the central data, portraying the distribution of injury severity as being higher than it was. A selection bias might exist due to the likelihood of more active and experienced athletes being drawn to the study. However, cross-referencing with the air ambulance database has helped mitigate this. It’s possible that participating athletes are more safety-conscious, potentially leading to higher usage of protective gear. Despite limitations as a small descriptive study, this article provides valuable insights into kiteboarding injury patterns and mechanisms.

Due to the low number of accidents identified in our study, the results must be interpreted with caution. However, we would like to emphasise our safety concerns in kiteboarding, illustrated by the risk of severe injuries, the high proportion of head injuries, and the low use of helmet and life vests in kitesurfing. To gather more knowledge about kiting injuries, it’s crucial to improve accident registration. Additional research is warranted concerning the consequences of kite-related injuries, particularly with regards to the level of medical attention required, duration of hospitalization, and the number of days of disability.

## Conclusions

Serious injuries occurred during kiteboarding. The injuries varied from minor lacerations to multitrauma. The majority of kiteboarders named operator error or lack of experience as the main reason for the accident. To reduce future injuries, participating in a kite course should be mandatory before kiteboarding. The kite course instructor should emphasise the importance in using helmet in snowkiting and both helmet and life vest in kitesurfing, in addition to the effects of wind strength and wind gust.

### Electronic supplementary material

Below is the link to the electronic supplementary material.

Supplementary Material 1

## Data Availability

The datasets supporting the conclusions of this article are included within the article.

## Abbreviations

REC West: Regional Committees for Medical and Health Research Ethics West
NACA: National Advisory Committee of Aeronautics score
ISS: Injury severity score
AIS: Abbreviated injury scale

## References

[1] Nickel C, Zernial O, Musahl V, Hansen U, Zantop T, Petersen W (2004). A prospective study of kitesurfing injuries. Am J Sports Med.

[2] Andreas Toverud sets record for the longest kite jump. http://www.surfertoday.com/kiteboarding/11799-andreas-toverud-sets-record-for-the-longest-kite-jump. Accessed June 28th 2023.

[3] Pikora TJ, Braham R, Hill C, Mills C (2011). Wet and wild: results from a pilot study assessing injuries among recreational water users in Western Australia. Int J Injury Control Saf Promotion.

[4] Bourgois JG, Boone J, Callewaert M, Tipton MJ, Tallir IB (2014). Biomechanical and physiological demands of kitesurfing and epidemiology of injury among kitesurfers. Sports Med (Auckland NZ).

[5] Moroder P, Runer A, Hoffelner T, Frick N, Resch H, Tauber M (2011). A prospective study of snowkiting injuries. Am J Sports Med.

[6] Driessen A, Probst C, Sakka SG, Eikermann C, Mutschler M. [Bilateral carotid artery dissection in a kite surfer by strangulation with the kite lines]. Der Unfallchirurg 2014. 10.1007/s00113-014-2641-0 25135706

[7] Durnford AJ, Harrisson SE, Eynon CA (2014). Kitesports: a new source of major trauma? Report of four cases and literature review. Trauma (United Kingdom).

[8] Exadaktylos AK, Sclabas GM, Blake I, Swemmer K, McCormick G, Erasmus P (2005). The kick with the kite: an analysis of kite surfing related off shore rescue missions in Cape Town, South Africa. Br J Sports Med.

[9] Spanjersberg WR, Schipper IB (2007). Kitesurfing: when fun turns to trauma-the dangers of a new extreme sport. J Trauma.

[10] Snowball Sampling Method.: Definition, Techniques & Examples. https://www.simplypsychology.org/snowball-sampling.html. July 7th 2023.

[11] Tryba M, Brüggemann H, Echtermeyer V (1980). [Klassifizierung Von Erkrankungen Und Verletzungen in Notartztrettungssystemen]. Notfallmedizin.

[12] Baker SP, O’Neill B, Haddon W, Long WB (1974). The injury severity score: a method for describing patients with multiple injuries and evaluating emergency care. J Trauma.

[13] Gennarelli TA, Wodzin E (2008). Abbreviated injury scale 2005: update 2008: Barrington.

[14] van Bergen CJ, Weber RI, Kraal T, Kerkhoffs GM, Haverkamp D (2020). Kitesurf injury trauma evaluation study: a prospective cohort study evaluating kitesurf injuries. World J Orthop.

[15] Rekand T (2012). The epidemiology of injury in hang-gliding and paragliding. Med Sport Sci.

[16] Kruger-Franke M, Siebert CH, Pforringer W (1991). Paragliding injuries. Br J Sports Med.

