# Supplementary material for: Kitesurfing and snowkiting injuries in Norway: a retrospective study
Source: BMC Sports Sci Med Rehabil. 2024 Jan 22;16:26. doi: 10.1186/s13102-024-00812-w (PMC10804644; doi:10.1186/s13102-024-00812-w)
Supplement: Supplementary file 1 — Supplementary Material 1 [file 13102_2024_812_MOESM1_ESM.docx]

| Additional file  The NACA scale; as used by the Norwegian Air Ambulance Service* | | |
| --- | --- | --- |
| NACA | **Description** | |
| 0 | No injury or disease. |  |
| 1 | Injuries/diseases without any need for acute physician care. | *E.g., transient hypotension and abrasions.* |
| 2 | Injuries/diseases requiring examination and therapy by a physician, but hospital admission is not indicated. | *E.g., moderate soft tissue injury and ruptured tendons.* |
| 3 | Injuries/diseases without acute threat to life, but requiring hospital admission. | *E.g.,* *cerebral concussion (unconscious < 15 min, no pathological neurology), large lacerations, open wounds with vascular or neurological injury, and fractures.* |
| 4 | Injuries/diseases that can possibly lead to deterioration of vital signs. | *E.g., cerebral concussion (unconscious > 15 min), fracture of tubular bone, several rib-fractures, and thoracic injury with unilateral haemo- or pneumothorax.* |
| 5 | Injuries/diseases with acute threat to life. | *E.g., large and complex fractures, several tubular bone fractures or single femur fracture, rib-fracture with respiratory distress, and cerebral concussion with anticipated increased intracerebral pressure.* |
| 6 | Injuries/diseases transported after successful resuscitation of vital signs. | *E.g., central nerve system injury affecting respiration or circulation, thoracic injury with respiratory distress or multiple fractures, and respiratory or cardiac arrest.* |
| 7 | Lethal injuries or diseases (with or without resuscitation attempts). |  |
| * This NACA scale is the version modified by Tryba et al. in 1980 for severity assessment in prehospital services.^11^ | | |
